# Monthly Summary

**Published:** 1884-12

**Authors:** 


### MONTHLY SUMMARY.

---

AMALGAM NOTES.—Dr Bogue at a recent meeting the New York Odontological Society, said:

"I must confess my disappointment when I hear gentlemen of The Odontological Society speak of squeezing their amalgam, and I am not surprised that any gentlemen who manipulates his amalgam this way should find it utterly unreliable.

"It is not much more than a month since one of my professional brethren said to me, 'What do you mean by weighing the component parts of your amalgam; I could not spend the time, how do you do it?' Now I supposed that every member of this Society knew a Fletcher Balance as well as I know a water jug. This gentlemen did not. I showed him how to use it and he acknowledged it was a good thing.

"I frequently see the the names of gentlemen belonging to the Odontological Society are given to the makers of amalgams, certifying this: 'Your amalgam is the best I have ever used,' etc. Either this means something or it means nothing. If he has carefully tested by mechanical and chemical tests, and by specific gravity, and knows whereof he affirms, that testimony ought to be valuable, but if he merely guesses and tells of some fillings he guesses have been in six months or guesses they have been in six years, and says they look well; if he rubs the amalgam in his hand and says' 'Yes, I will give you a certificate,' it does not show that in this 'district school' we are learning a great deal."—*Archives of Dentistry.*

---

FOR SENSITIVE DENTINE.—"Dr. T. A, Robinson, it will be seen in our report of the American Dental Association recommends equal parts, by weight, of carbolic acid and caustic potash ground together.

He applies in small portions to the cavity, repeating it if necessary. He also finds it good as an application to aching exposed pulps preparatory to treatment. It sometimes gives a little pain—but not severe. He modified it, occasionally, by adding oil of cloves, or creasote, as in his judgment the case might require. He had used it for about twelve years with increasing satisfaction. He thinks it seldom needful to destroy exposed pulps.—*Items of Interest.*

---

CONSOLIDATION.—It is not often the case, when one gets married, that the event is spoken of as a "death," or that one of the parties to the transaction is referred to as "deceased," etc. Some of our contemporaries, in referring editorially to the recent *consolidation* of the ARCHIVES and the *New England Journal of Dentistry*, either misunderstand a plain statement or wish to mislead the profession. To record, without modification, of the *New England Journal of Dentistry* that it is "no more" is a mistake. To state, in an unqualified manner that it has "succumbed" is—if the word is used intentionally and with an intelligent appreciation of its true meaning—an egregious mistake.

*The New England Journal* protests that it is not ready for an obituary notice; that it is not "deceased." Neither have its editors "dried" their "pens." "Self denying labors have not ceased" and "weary brains" refuse the "rest" to which they are consigned.

A simple change in names or, other words, "getting married" does not warrant or justify a friendly cotemporary in the use of such terms as "deceased," "succumbed," etc. There is nothing of the kind involved in the transaction, and the editors of *The New England Journal* decline to be buried by means of sugar coated obituary notices. They have other "fallacies" to maintain and rejoice that the new arrangement affords a larger field in which to disseminate them.—*ArchivesofDen.*

---

TETANUS TRHATED BY ETEER SPRAY.—Dr. Boutellier has communicated to the *Progress Medical* a case of traumatic tefanus and another of cholera treated successfully by him, with the application of ether spray to the spinal column. A man whilst driving struck his horse with the end of the reins, and received the *contaecoup* upon the third finger. A small wound, which bled profusely, was the result, and a week afterwards cicatrization was complete. However, his business called him out again, and he had to make a long journey when the weather was very cold On returning he felt great oppression, and two days afterwards he could with difficulty open his mouth, and in the night of the same day he was seized with constriction in the chest every five minutes. When Dr. Boutellier arrived, he found the patient motionless in his bed, the jaws tightly shut, and the lateral muscles of the neck contracted, episthotones well marked. A purgative was ordered, and pulverization of ether on the spine every hour for five minutes at a time. The next day the patient was notably better. Calabar bean according to the formula of Watson was also administered. From this time the attacks diminished, and the ether spray was kept up every two hours for a few days longer, when the patient was considered entirely convalescent. The second case was no less striking. A boy, æt. 11, was suffering from well marked chorea, for which he was treated with opium, bromide of potassium, arsenic, and calabar bean, for five weeks without the slightest advantage. It was then that the author thought of the treatment of Lubelski. Ether spray was applied to the spine morning and evening from three to five minutes at a time. The second day the boy was better, and at the end of a fortnight of the treatment all chronic symptoms had ceased, and never returned.

SULPHO-CARBOL, THE NEW ANTISEPTIC.—From the *London Med. Times* we learn that M. Pierre Vigier, the able writer of the "Pharmaceutical Contributions" in the *Gazette Hebdomadaire*, giving an account (in the number for Aug. 8) of a paper on a new antiseptic, read at the Societe de Biologie, by M. Ferdinand Vigier, a *pharmacien* of Paris, observes that the fact of its author being his near relative is an additional security for his examining the pretensions of the substance to an important position. The substance in question is orthoxyphenic-sulphurous acid, which may be more conveniently named *sulpho-carbol*, as more simply indicating a combination of sulphuric acid or phenic acid. The antiseptic and antifermentative properties of this compound are remarkable, and it has the advantage over carbolic acid of being soluble in water in all proportions, and of being neither poisonous nor caustic it is a syrup, rose-colored liquid, of a pungent odor, but no wise disagreeable in solution. It is votallized in a water-bath, and may be used for fumigation. It was discovered in 1841 by Laurent, and has since then engaged the attention of many chemists. Recently its properties have been investigated by M. F. Vigier, with the assistance and in the laboratory of M. Laborde, and its great antiseptic powers have been duly demonstrated. It may be given internally also, in syrup and water, in doses of from one to five grammes daily. Indeed, being an inoffensive product, its doses may be increased *ad libitum*, which of course is not the case with carbolic or salicylic acids.—*Med. and Sur. Reporter.*

